# Relationship Between Waist Circumference, Epicardial Fat Thickness, and Genders

**DOI:** 10.21470/1678-9741-2020-0306

**Published:** 2021

**Authors:** Fatih Aksoy, Serdar Güler

**Affiliations:** 1 Department of Cardiology, Süleyman Demirel Faculty of Medicine, Isparta, Turkey.; 2 Department of Cardiology, Acıpayam State Hospital, Denizli, Turkey.

We would like to thank Engin M.^[^^1^^]^ for his kind interest and considerations on our article^[^^2^^]^. He pointed out that the waist circumference (WC) should be evaluated separately for men and women^[^^1^^]^. This is indeed a valid suggestion. In our study, we did not analyze WC separately for men and women, we evaluated the total population. We aimed only to assess epicardial fat thickness (EFT) and the CHA_2_DS_2_-VASC score including cardiovascular risk factors.

Abdominal obesity (AO) is associated with an increased risk of cardiovascular disease. WC, which is an indicator of AO, has been shown to correlate with EFT^[^^3^^]^. In the study by Jeong et al., similarly to ours, no separate assessment was made between the genders^[^^3^^]^. In our study, it has been shown similar results. According to the Jeong et al. suggestion, when the genders were evaluated separately, it was found that both men and women were similarly correlated (r=0.218, *P*=004 and r=0.216, *P*=0.05, respectively)^[^^3^^]^. Our results showed that when the gender was evaluated separately, the correlation coefficient did not change much, but there could be a change in significance. This situation can be explained by using numerical variables instead of categorical variables, such as gender, while calculating the Spearman's rank correlation coefficient and performing Pearson's correlation analysis^[^^4^^]^.

We therefore agree that larger-scale studies should be conducted to clarify the difference of EFT and WC between the genders. Consequently, gender-based studies in the following years may bring a more explanatory view to this situation.
